# On the mechanism of calcium‐dependent activation of NADPH oxidase 5 (NOX5)

**DOI:** 10.1111/febs.15160

**Published:** 2019-12-20

**Authors:** Elisa Millana Fañanás, Sofia Todesca, Alessandro Sicorello, Laura Masino, Petr Pompach, Francesca Magnani, Annalisa Pastore, Andrea Mattevi

**Affiliations:** ^1^ Department of Biology and Biotechnology “Lazzaro Spallanzani” University of Pavia Italy; ^2^ UK Dementia Research Institute at King's College London UK; ^3^ The Wohl Institute at King's College London UK; ^4^ The Crick Institute London UK; ^5^ Institute of Biotechnology Czech Academy of Sciences Vestec Czech Republic; ^6^ Institute of Microbiology Czech Academy of Sciences Prague Czech Republic

**Keywords:** calcium activation, EF‐hands, enzyme, NMR, structure

## Abstract

It is now accepted that reactive oxygen species (ROS) are not only dangerous oxidative agents but also chemical mediators of the redox cell signaling and innate immune response. A central role in ROS‐controlled production is played by the NADPH oxidases (NOXs), a group of seven membrane‐bound enzymes (NOX1‐5 and DUOX1‐2) whose unique function is to produce ROS. Here, we describe the regulation of NOX5, a widespread family member present in cyanobacteria, protists, plants, fungi, and the animal kingdom. We show that the calmodulin‐like regulatory EF‐domain of NOX5 is partially unfolded and detached from the rest of the protein in the absence of calcium. In the presence of calcium, the C‐terminal lobe of the EF‐domain acquires an ordered and more compact structure that enables its binding to the enzyme dehydrogenase (DH) domain. Our spectroscopic and mutagenesis studies further identified a set of conserved aspartate residues in the DH domain that are essential for NOX5 activation. Altogether, our work shows that calcium induces an unfolded‐to‐folded transition of the EF‐domain that promotes direct interaction with a conserved regulatory region, resulting in NOX5 activation.

## Graphical abstract

The N‐terminal domain (EF) of NADPH oxidase 5 contains four EF‐hand motifs and is the main player in this enzyme activation. In the absence of calcium, EF‐hands 3 and 4 are unfolded and the domain remains separated from the catalytic dehydrogenase domain (DH). In the presence of calcium, EF‐hands 3 and 4 acquire a structure and bind to the DH domain, activating the electron flow and production of reactive oxygen species.

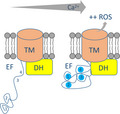

**Abbreviations**
CD: circular dichroism
csNOX5: NOX5 from *Cylindrospermum stagnale*
DH: dehydrogenase
DUOX: dual oxidase
GL: gel filtration
hNOX: human NOX
NMR: nuclear magnetic resonance
NOX: NADPH oxidase
p.p.m.: part per millions
ROS: reactive oxygen species
SEC‐MALLS: size‐exclusion chromatography/multiangle light

## Introduction

Radical oxygen species (ROS) have a dual role: They are the cytotoxic molecules at the heart of oxidative stress but are also critical chemical agents in immune response as well as in signaling pathways that mediate cell growth, differentiation, and death 1, 2, 3. Main players in the ROS‐mediated processes are members of the NADPH oxidase (NOX) family. NOX enzymes share a catalytic core formed by six transmembrane helices, with two noncovalent heme molecules, followed by a C‐terminal dehydrogenase (DH) domain that binds NADPH and FAD. Electrons donated by cytosolic NADPH are sequentially transferred to FAD, heme, and lastly to an O_2_ molecule to produce O_2_^‐^ radicals and/or H_2_O_2_ at the opposite side of the cell membrane 4.

Humans contain a total of seven NOX enzymes: NOX1‐5 and DUOX1‐2. The distinction among NOX enzymes stems from their specific regulatory mechanisms. NOX1‐3 function in complex with the membrane protein p22phox and are regulated by multiple intracellular proteins. NOX4 forms a complex with p22phox but has no known enzymatic regulatory mechanism as it is the only constitutively active human NOX (hNOX) 5. NOX5 is a monomeric protein that contains a regulatory calmodulin‐like EF‐hand domain, a transmembrane domain, and the catalytic DH core. Activation of NOX5 is calcium dependent 6. Other proteins, such as calmodulin, Hsp90, caveolin‐1, and the tyrosine kinase c‐Abl, have been reported to modulate NOX5 activity but are not strictly required for its functionality 7, 8, 9, 10. DUOX1‐2 has a mixed regulation, as they possess a regulatory EF‐domain but also need oligomerization with their accessory proteins DUOX‐A1/2 11.

NOX5 is expressed in several human tissues, including lymphoid testis, endothelial cells, and smooth muscle. Recent data implicate NOX5 in cell transformation and cancer 12, 13. Yet, NOX5 is absent in rodents which has hampered murine model studies 13. The evolutionary process by which rodents lost this enzyme is unknown but data suggest that NOX5 functions are replaced by other family members 13. The presence of NOX5 is not only confined to vertebrates: NOX5‐like proteins are also found in vertebrates, plants, fungi, protists, and cyanobacteria. The wide distribution of NOX5‐homologues across different kingdoms has thus suggested that NOX5 is the most ancient NOX‐family member 14. In plants, NOX5‐like enzymes, called respiratory burst oxidase homologue, have received special attention because of their involvement in tissue development and response to environmental stimuli. Importantly, all these functions are calcium regulated 15.

In a previous paper, we reported the first crystallographic structural model of the catalytic core of a NOX protein 4. The structural analysis was performed using the catalytic core of NOX5 from *Cylindrospermum stagnale* as a model system (csNOX5). This cyanobacterial NOX5 shares 40% sequence identity to hNOX5. The crystal structures of the DH (residues 413–693) and transmembrane (residues 209–412) domains of *C. stagnale* NOX5 confirmed the postulated mechanism of electron transfer and unveiled both the architecture of the O_2_‐binding site and the structural motifs that regulate ROS generation. While these results have appreciably increased our knowledge on the ROS‐producing catalytic activity of NOXs, the structural basis of NOX5 regulation by calcium remains mostly unknown since the crystal structure of the EF‐domain has not been obtained so‐far.

The EF‐domain belongs to the superfamily of EF‐hand proteins. In the hNOX5 isoform β, the EF‐domain is composed of 161 N‐terminal amino acids linked to the transmembrane domain by a short basic sequence that regulates the interaction with the membrane 16. As in many EF‐hand proteins, the EF‐domain of NOX5 is subdivided into N‐ and C‐lobes, each containing two EF‐hand motifs. The N‐lobe contains EF‐hands 1‐2 and has lower calcium affinity (*K*_d_ = 15–20 µm measured in hNOX5), while the C‐lobe comprises EF‐hands 3‐4 and features a fivefold higher calcium affinity (*K*_d_ = 3.8 µm in hNOX5) 17. The EF‐domain regulates NOX5 activity by interacting with the DH‐domain through a segment called regulatory EF‐binding domain 16. In the crystal structure of the *C. stagnale* DH‐domain, this region (residues 611–634) is disordered 4.

Here, we describe a structural characterization of the EF‐domain of hNOX5 (residues 1–161) carried out by different but complementary techniques ranging from CD and size‐exclusion chromatography/multiangle light scattering (SEC‐MALLS) to NMR and mutagenesis. We explored the interactions between the EF‐hand and DH‐domains to better understand the calcium‐dependent regulation of NOX5. We discovered that calcium induces a change in the overall conformation of the EF‐domain. We also found that the C‐lobe is the main player in regulating the catalytic domain of the enzyme. Based on these results, we propose that the C‐lobe of the EF‐domain acquires a folded and ordered structure upon calcium binding, and as a consequence, it is able to bind the DH‐domain, triggering enzyme activation.

## Results

### The apo EF‐domain of NOX5 is intrinsically unfolded but with residual secondary structure

**Figure 1:**
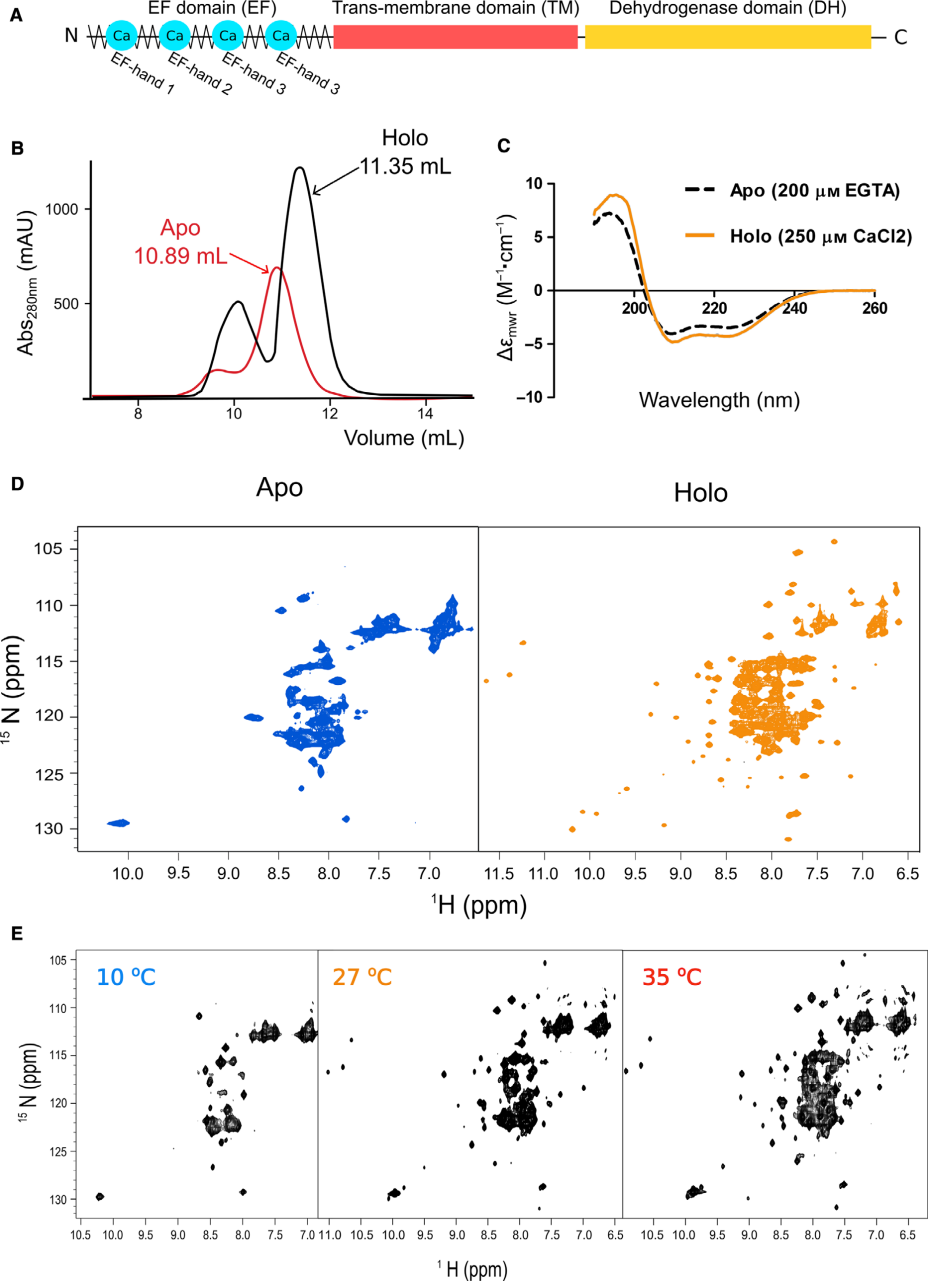
The EF‐domain of hNOX5 undergoes a large structural transition upon calcium binding. (A) NOX5 comprises three domains: The EF‐domain with four EF‐hand motifs (EF), the transmembrane domain, and the DH domain. (B) Size‐exclusion chromatography (Superdex‐75 10/300 column; GE Healthcare) elution profiles in presence or absence of calcium present different behavior, suggesting a more globular shape in the holo state. (C, D) CD spectra, and ^1^H‐^15^N HSQC spectra of the apo (calcium‐free) and holo (calcium‐bound) forms of the EF‐domain of hNOX5. The apo protein contained 200 µm EGTA, while the holo state was measured in the presence of 250 µm CaCl_2_, at 27 °C. Far‐UV‐CD measurements were done in duplicate. The CD spectra showed a lower secondary content in the apo state, consistently with the NMR, where there is a clear lack of proton dispersity (left) with respect to the defined resonances of the holo state (right). (E) Quality of the ^1^H‐^15^N HSQC NMR spectrum of the human holo EF‐domain is poor at low temperatures (10 °C) and improves at 27 and 35 °C.

We attempted production of the recombinant EF‐domain working in parallel with csNOX5 and hNOX5 with the aim of gaining insights into NOX5 regulation. The recombinant EF‐domain from csNOX5 (residues 1–180) turned out to be unstable and mostly insoluble (data not shown). By contrast, the EF‐domain from hNOX5 (residues 1–161, isoform β) (Fig. 1A) could be expressed and successfully purified as a stand‐alone soluble protein with yields as high as 6 mg·L^−1^ of bacterial culture. Size‐exclusion chromatography showed that the EF‐domain of hNOX5 has a different behavior in the presence and in the absence of calcium (Fig. 1B): The calcium‐free (apo) domain eluted at earlier volumes than the calcium‐bound (holo) protein, suggesting that the holo domain has a smaller and more globular shape in agreement with previous fluorescence studies 6. To better characterize the fold of the EF‐domain, we compared the far‐UV CD of the apo and holo proteins. Both spectra had all the features typical of helical proteins with two distinct negative minima around 208 and 222 nm (Fig. 1C). Spectral deconvolution provided an estimate of the helical content of ca. 25% for the apo protein. The intensity of the band at 222 nm in the spectrum of the holo state is 15% deeper than that of the apo protein (Table 1). This indicates that the protein fold is stabilized by calcium binding but the apo EF‐domain retains residual secondary structure also in the absence of the bound cations.

**Table 1:** Intensity of the CD band at 222 nm for the apo and holo states of the EF‐domain of hNOX5. The measurements were done in duplicates

	Δε_mwr_ at 222 nm (M^−1^·cm^−1^)		
	Apo	Holo	Increase (%)
Full‐length	−3.44	−4.28	14.7
N‐terminal lobe	−2.71	−4.00	12.2
C‐terminal lobe	−4.5	−4.42	17.7

The hNOX5 EF‐domain was further studied by NMR. The ^1^H‐^15^N HSQC spectra of the apo and holo‐forms were markedly different: The spectrum of the apo protein had a poor spectral dispersion with all the HN‐N connectivities overlapping within a few p.p.m. The holo EF‐domain had instead an excellent dispersion indicating that the protein becomes structured upon calcium binding (Fig. 1D). The quality of the spectrum of the holo EF‐domain was maximal at room temperature (27° C) as previously reported for other proteins 18 but was increasingly impoverished when lowering the temperature because of slower tumbling (Fig. 1E). The apparent discrepancy between the CD and NMR results could easily be resolved considering that CD detects also transient secondary structure whereas NMR chemical shifts report on the persistence of the protein in a defined tertiary environment. These results supported the conclusion that the EF‐domain is mostly unstructured in the absence of calcium, though retaining residual secondary structure. The EF‐domain acquires tertiary structure when loaded with calcium.

### The regulatory domain of NOX5 contains four active calcium‐specific EF‐hands

**Figure 2:**
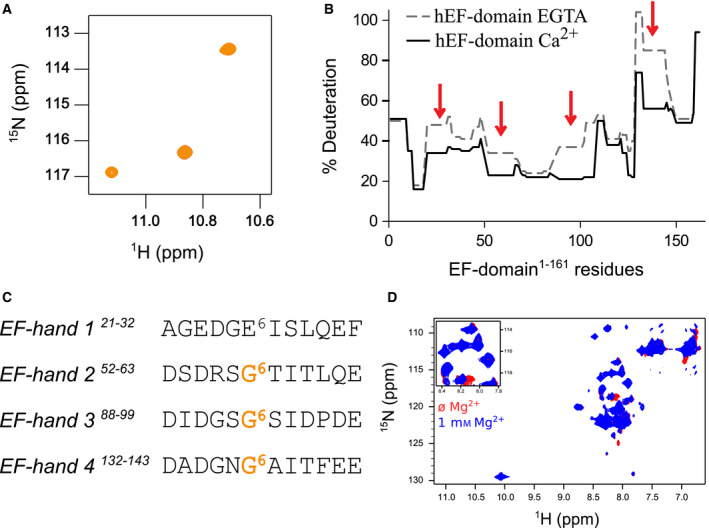
The regulatory domain of NOX5 contains four active calcium‐specific EF‐hands. (A) In the holo state, the ^1^H ^15^N HSQC spectra display three resonances at the proton frequencies 11.2, 10.86, and 10.705 p.p.m. likely corresponding to Gly57, Gly93, and Gly137. (B) Comparison of HDX performed with the purified hEF domain for the apo‐ and the holo‐form, independently. The overlap of both results shows four regions where the presence of calcium induced a reduction of the deuteration level, after 20 seconds of reaction. These four regions correspond to the predicted four EF‐hand motifs. (C) Sequences of the four EF‐hands motifs of hNOX5. EF‐hands 2, 3, and 4 have a glycine in position 6, while EF‐hand 1 contains glutamate. (D) Overlap of the ^1^H‐^15^N HSQC spectra measured with or without magnesium demonstrates that magnesium does not induce the transition to the holo conformation, contrary to calcium (Fig. 1C).

With the first experiments showing a clear conformational transition, we further studied the effect of calcium binding on the hNOX5 EF‐domain by a combination of techniques. Calcium titration induced the appearance in the ^1^H‐^15^N HSQC NMR spectra of three resonances at ~11 p.p.m. and ~115 p.p.m. in the proton and nitrogen dimensions respectively (Fig. 2A). These chemical shifts are characteristic of EF‐hand proteins and correspond to the conserved glycines that occupy position 6 of the canonical binding loops 19. In addition, we observed resonances at 9.2–10.2 p.p.m. and 126–130 p.p.m. These likely correspond to the resonances of the amides in the canonical position 8 of the calcium‐binding loops and appear only when calcium is bound 20. Thus, NMR confirmed that at least three EF‐hands of NOX5 bind calcium. These are very likely the canonical 2, 3 and 4 EF‐hands whereas the noncanonical EF‐hand 1 does not contain a diagnostic glycine in position 6 of the loop. As for the fourth potential calcium‐binding site, we referred to hydrogen‐deuterium exchange methods. Comparison between the calcium‐loaded and calcium‐free (in the presence of the chelator EGTA) protein forms indicated four main regions where calcium binding led to a significantly lower degree of deuteration (Fig. 2B). This phenomenon, called protection, reflects an increase compaction, possibly caused by binding of calcium. Indeed, the four protected regions perfectly overlapped with the four predicted EF‐hands motifs, validating the notion that the EF‐domain of NOX5 contains four calcium‐binding sites (Fig. 2C).

Since some canonical EF‐hands can also be activated by Mg^2+^, whose concentration in cells is higher than that of calcium (0.5–5 mM) 21, we explored the specificity of the hNOX5 EF‐domain for calcium. ^1^H‐^15^N HSQC spectra recorded in the presence/absence of an excess of Mg^2+^ showed only minor differences and no conversion to the holo‐form (Fig. 2D). Therefore, NOX5 binds calcium preferentially.

### The C‐lobe of the EF‐domain is the main responsible of the unfolded apo state

**Figure 3:**
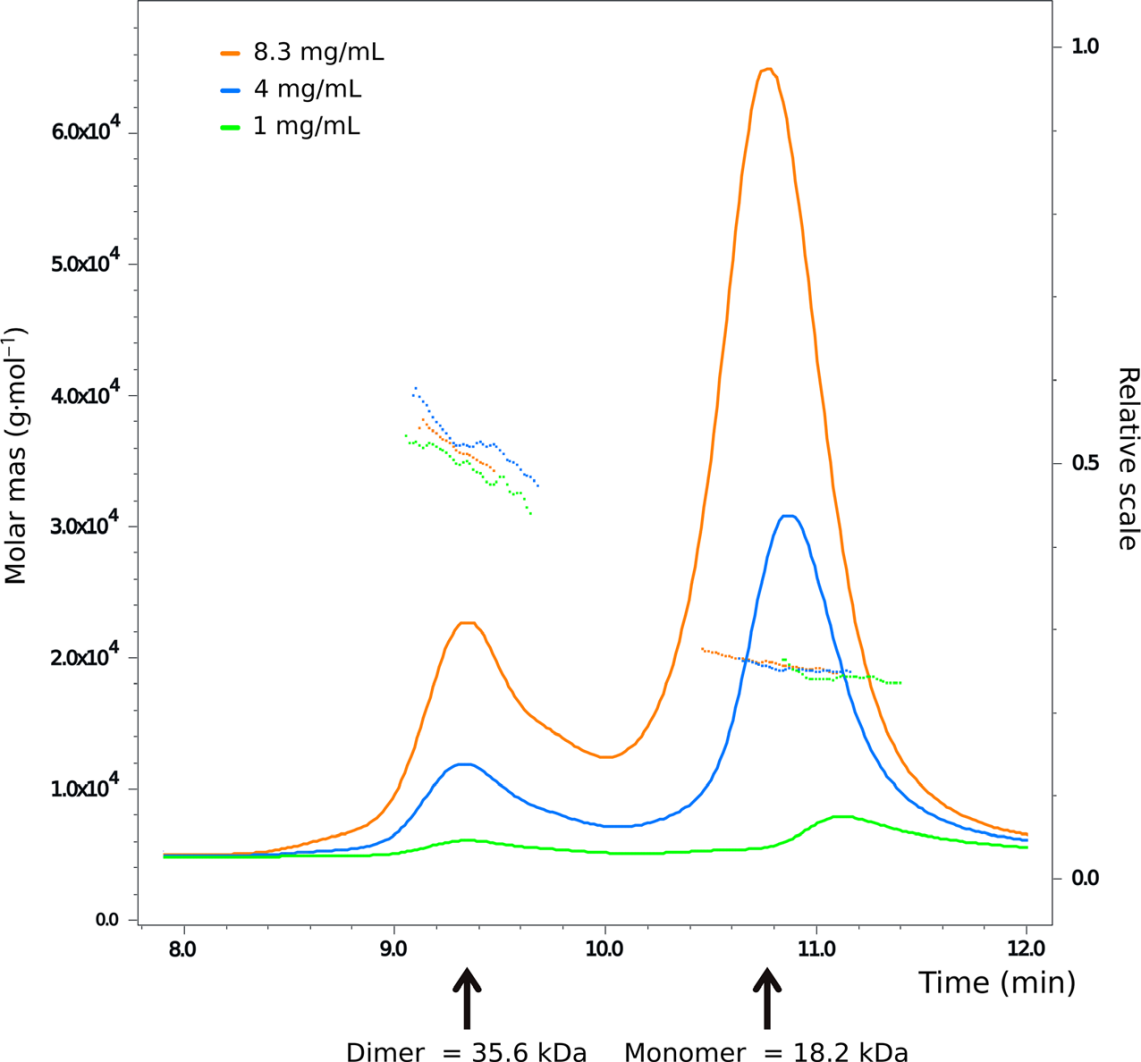
SEC‐MALLS analysis shows that the EF‐domain of hNOX5 is mostly monomeric in solution, with a 15–20% dimerization. Samples (100 μL) were applied to a Superdex 200 10/300 GL column mounted on a Jasco HPLC equilibrated in 50 mM Hepes buffer at pH 7.0, 25 mM NaCl, 0.5 TCEP, at a flow rate of 1 mL·min^−1^. Measurements were recorded using a DAWN HELEOS laser photometer and an OPTILAB‐rEX differential refractometer (ΔRI) (dn/dc = 0.186).

Attempts to assign the spectrum of the holo‐form of the EF‐domain failed because the HNCA and HNCACB spectra were almost empty (data not shown). To explain this result, we ran SEC‐MALLS, a sensitive technique able to detect the state of oligomerization of proteins. We observed that, at the protein concentrations under study, the EF‐domain is mostly monomeric but in the presence of a minor population of dimers (12–15%; Fig. 3). The low signal‐to‐noise ratios observed by NMR could thus be the consequence of an intermediate exchange regime between the monomer and the dimer or of an adverse dynamic regime.

**Figure 4:**
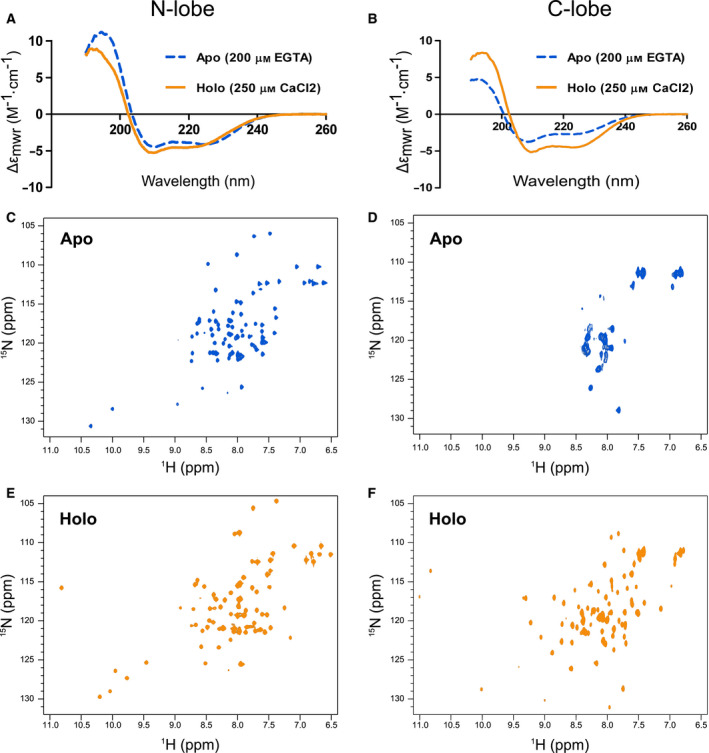
CD and NMR characterization of the N‐ and C‐lobes of hNOX5 EF‐domain. (A, B) CD spectra of the two proteins. The apo proteins (blue discontinuous line) contained 200 µm EGTA, while the holo proteins (orange continuous line) were incubated in a solution containing 250 µm CaCl_2_. Differences between apo and holo are mainly visible for the C‐lobe. Far‐UV‐CD measurements were done in duplicate. (C, D) The ^1^H‐^15^N HSQC spectrum of the N‐lobe apo state has a good dispersity in contrast to the poor dispersity of the apo C‐lobe protein. (E, F) Both N‐ and C‐lobes display an excellent dispersion in the holo state, leading to conclude that both are highly structured in presence of calcium. All proteins were in 50 mM Hepes buffer at pH 7.0, 25 mM NaCl, 0.5 TCEP. The measurements were carried out at 27 °C.

To circumvent the problem, we produced the two lobes of the EF‐domain individually. They could be expressed and purified as stand‐alone proteins without difficulties. The far‐UV CD spectra of these fragments had all the features typical of helical proteins similar to those observed for the parent domain (Fig. 4A,B). The C‐lobe had larger differences between holo and apo forms, with 18% of intensity increase at 222 nm for the holo‐form, while the holo N‐lobe had an increase of only 12% (Table 1). The minimum at 208 nm in the spectrum of apo C‐lobe is also shifted toward 200 nm indicating a higher content of random coil. NMR studies clarified the meaning of these observations: The apo N‐lobe featured a well‐resolved spectrum, whereas the peaks in the spectrum of apo C‐lobe were highly overlapping (Fig. 4C,D). Conversely, the spectra of the holo‐forms of both proteins had an excellent dispersion with sharp peaks showing that both lobes are monodispersed and highly soluble in the presence of calcium (Fig. 4E,F). Collectively, the CD and NMR data led to a main conclusion: The N‐lobe is highly structured both in the apo and in the holo states while the C‐lobe becomes fully structured only in the presence of calcium.

**Figure 5:**
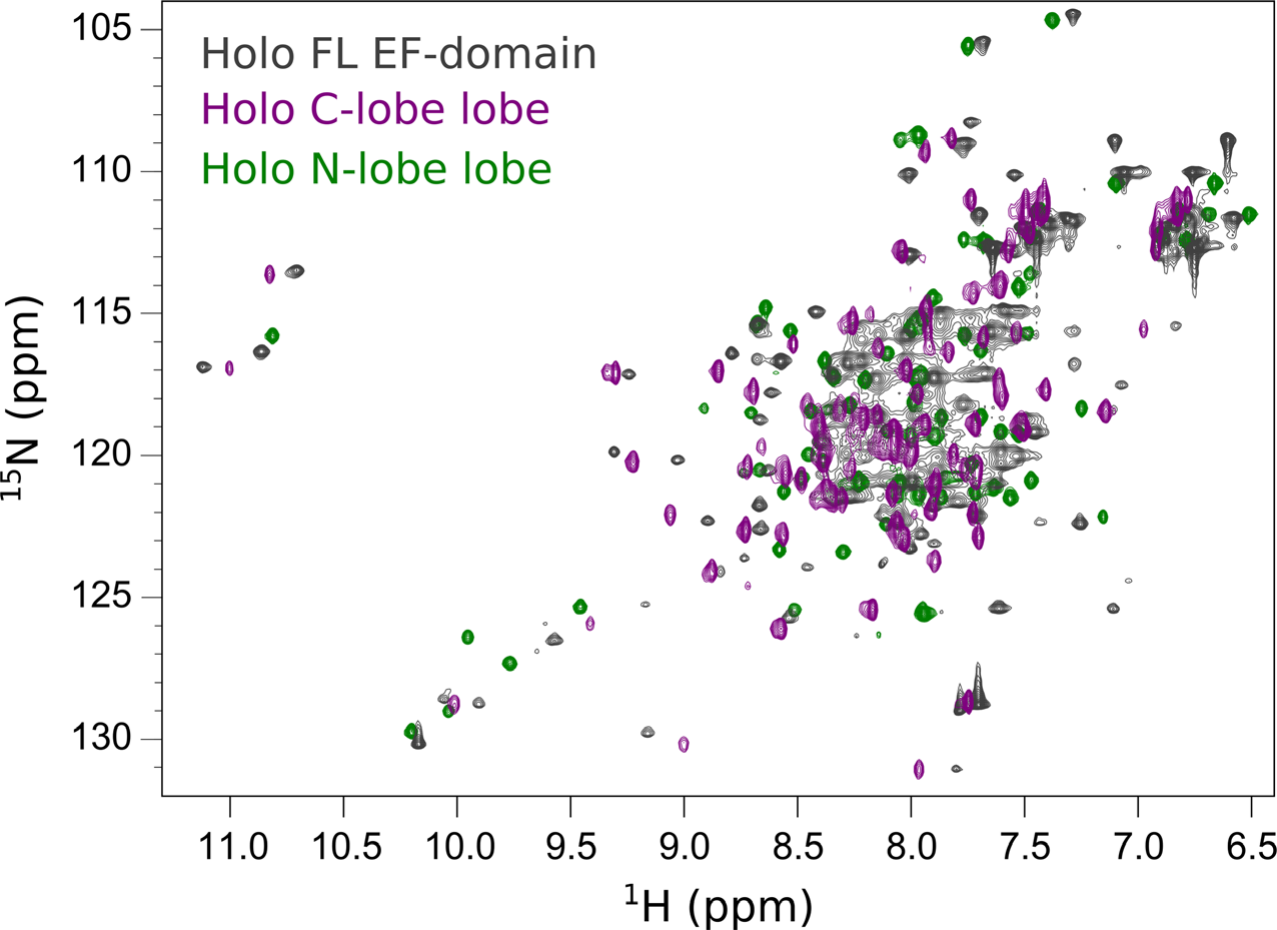
Lack of additivity of the two halves of the EF‐domain. The spectrum of the full‐length EF‐domain (gray) does not match the spectrum calculated by the addition of the ^1^H‐^15^N HSQC spectra at 800 MHz of the N‐ (green) and C‐ (purple) lobes. All proteins were in 50 mM Hepes buffer at pH 7.0, 25 mM NaCl, 0.5 TCEP. The measurements were carried out at 27 °C.

A typical feature of calmodulin‐like proteins is that the two globular lobes often behave as semi‐independent proteins so that the presence of one does not appreciably induces chemical shift variations in the other spectrum. As a consequence, superposition of the NMR spectra of the two isolated lobes leads to a spectrum that is almost indistinguishable from the spectrum of the whole EF‐domain since the two lobes do not influence each other. In agreement with previous tryptophan fluorescence data 6, this was not the case for the hNOX5 EF‐domain: The spectrum of the full‐length domain was evidently different from the spectrum obtained by superimposing the spectra of the individual N‐ and C‐lobes (Fig. 5). We also found that titrations of the labeled N‐lobe with the unlabeled C‐lobe and vice versa did not show detectable interactions (data not shown). This implies that their affinity is too low to interact even at the relatively high protein concentrations employed in the NMR experiments. A reasonable conclusion is that the two lobes must be linked together to interact.

### The C‐lobe of the EF‐domain is the main responsible for binding to the DH‐domain

**Figure 6:**
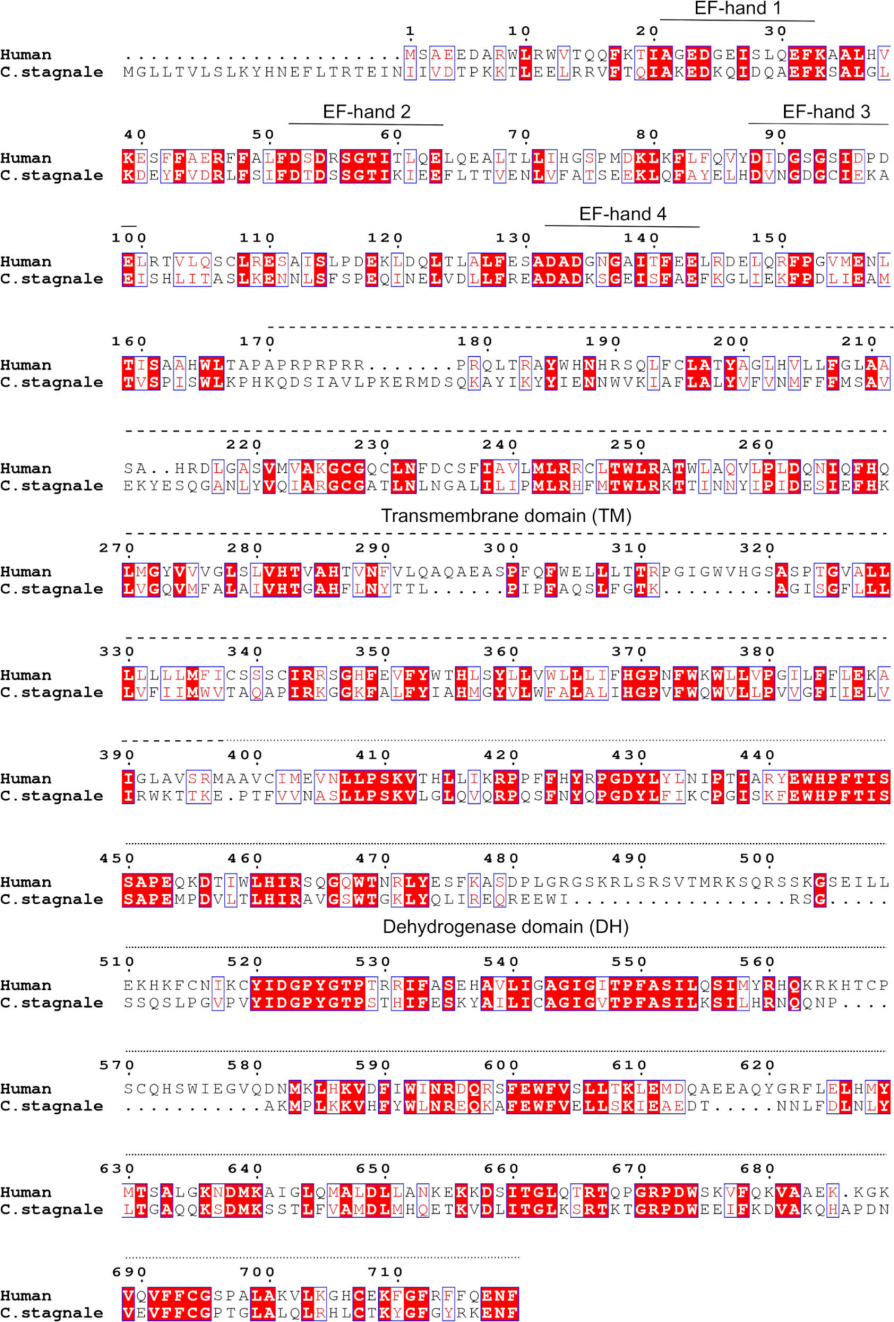
Alignment of human and *C. stagnale* NOX5 sequences prepared using ESPript3. UNIPROT sequences codes: csNOX5 K9WT99-1; hNOX5 Q96PH1-4.

**Figure 7:**
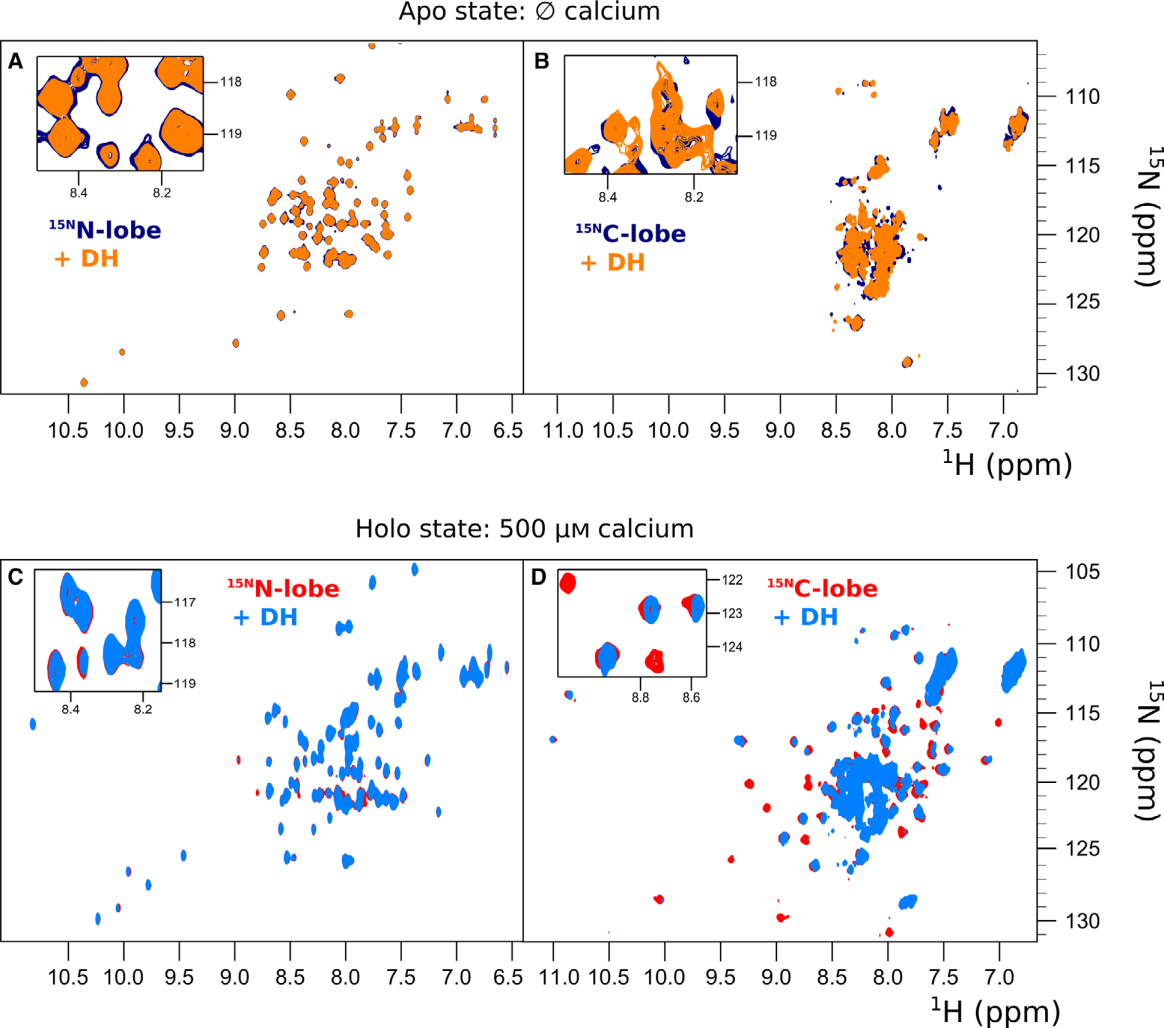
The C‐lobe of the EF‐domain is the main element for the binding to the DH‐domain. (A, B) In the absence of calcium, none of the lobes of the human EF‐domain present clear spectral differences when a twofold excess of the *C. stagnale* DH‐domain was present. (C, D) In the presence of calcium, the spectrum of the N‐lobe remains unperturbed by the DH‐domain, while the C‐lobe is highly affected. The intensities of several resonances are decreased or lost as the result of a weak yet clear interaction.

**Figure 8:**
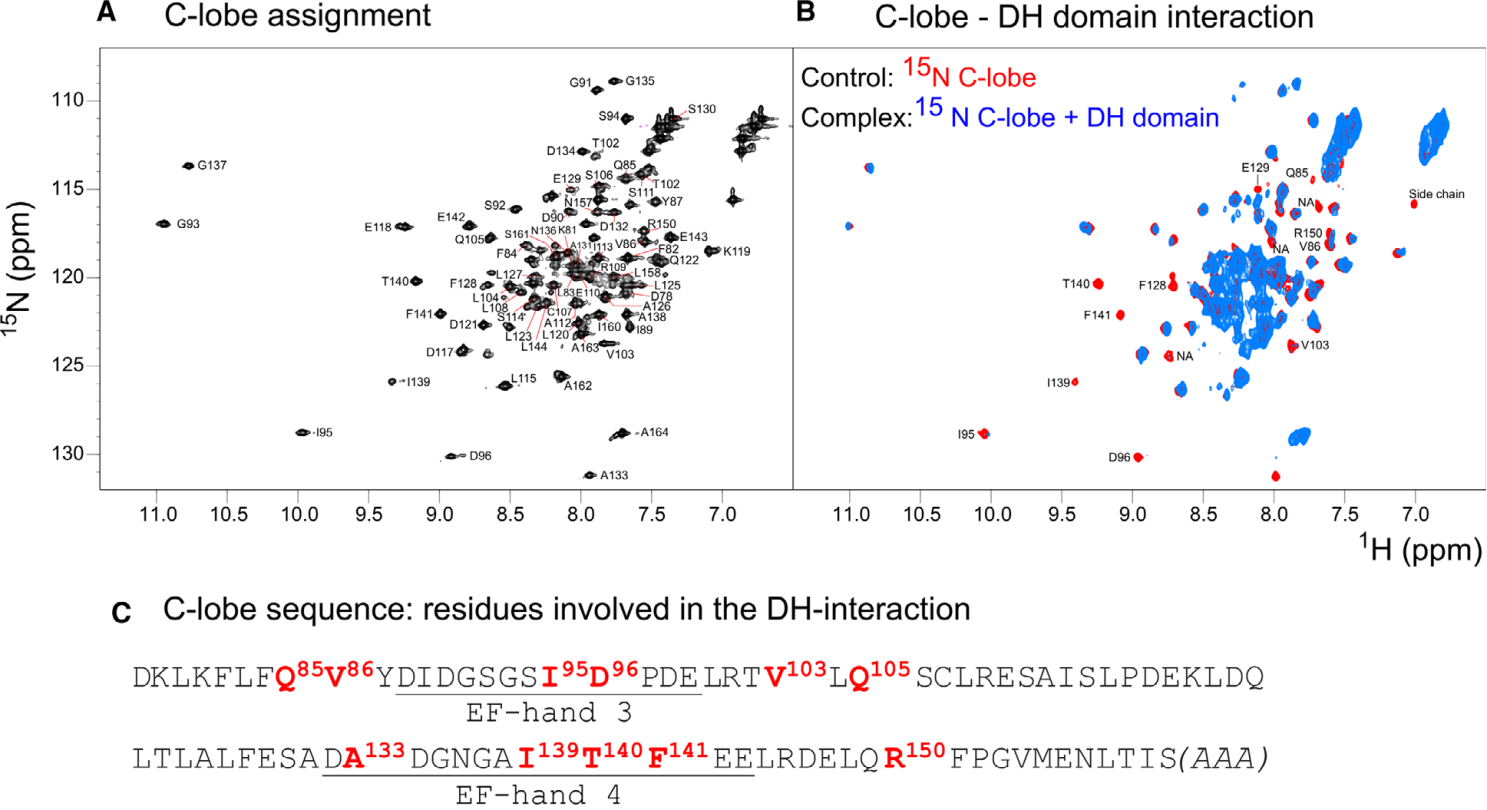
Identification of the residues involved in the interaction between the C‐lobe of the human EF‐domain and the DH‐domain of csNOX5. (A, B) NMR spectra and residue assignment of the C‐lobe in absence and presence of the DH‐domain. (C) The interacting residues (red) are mainly located in the loops of EF‐hands 3 and 4 (underlined in the sequence). The terminal alanines (in brackets) are an addition due to cloning needs.

We took advantage of the excellent quality of the spectra of the individual lobes to gain preliminary information on the interaction between the EF‐ and DH‐domains. We titrated the labeled individual lobes with the unlabeled DH‐domain. We used the DH‐domain from csNOX5 because, in our hands, the domain from hNOX5 could not be produced in sufficient quantities and at the purity required for a biophysical characterization 4. The sequences of the human and *C. stagnale* DH‐domain share anyway close similarity (45% identity) which suggests that the *C. stagnale* domain could be used, in a first instance, as a substitute for the human protein (Fig. 6). In the absence of calcium, the addition of the DH‐domain did not produce appreciable variations of the spectra of either EF‐domain lobes (Fig. 7A,B). Likewise, the spectrum of the N‐lobe was unaffected by the DH‐domain also in the presence of calcium. Thus, the N‐lobe does not seem to interact with the catalytic domain or, if it does, the affinity is so low that the interaction is lost when the domains are studied independently. Conversely, the C‐lobe spectrum was greatly and evidently affected by the DH‐domain in the presence of calcium (Fig. 7C,D). No new resonances appeared but the intensity of many resonances significantly decreased or disappeared. These features are indicative of a weak but clear association. Spectral assignment, completed at 80%, allowed us to map the interaction on the surface of the C‐lobe (Fig. 8A,B). The residues affected by the interaction with the DH‐domain are grouped in or around the predicted calcium‐binding loops (Fig. 8C). While these results need to be confirmed with a homologous system containing proteins from the same species, this is a preliminary indication that the C‐lobe is the main player in the interaction of the EF‐domain with the catalytic domain under calcium‐activated conditions.

### Two aspartates in the catalytic domain are indispensable for the EF‐domain to activates NOX5

**Figure 9:**
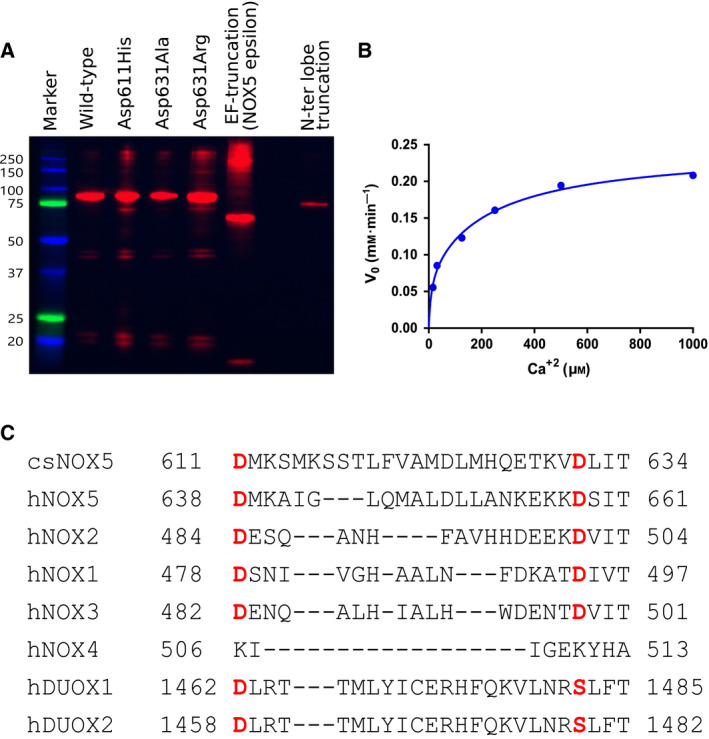
Mutagenesis targeting the EF‐domain binding area. (A) Western blot analysis of the semi‐purified membranes of the csNOX5 mutants indicated that all variants had expression levels similar to the wild‐type, except for the N‐lobe truncation that had a lower expression. Because the extract is not pure protein, these methods give only a comparative impression of the relative expression. The marker used was Precision Plus Protein™ Dual Color Standards (Bio‐Rad). Molecular weights are in kDa. The high‐molecular weight species are most‐likely protein aggregates. The Western blot was repeated in triplicates. (B) The csNOX5 wild‐type activity in response to increasing concentrations of calcium was measured in membrane fractions following the reduction of cytochrome c (Sigma). At 1 mM CaCl_2_ plateau was reached. Activity measurements were carried out in triplicate. (C) Sequence comparison of the regulatory ‘region EF‐binding domain’ on the DH domain of csNOX5 and hNOX5 with the corresponding protein regions of hNOX1‐4 and human DUOX1‐2. UNIPROT codes: csNOX5 K9WT99-1; hNOX5 Q96PH1-4; hNOX1 Q9Y5S8-1; hNOX2 P04839; hNOX3 Q9HBY0-1; hNOX4 Q9NPH5-1; hDUOX1 Q9NRD9-1; hDUOX2 Q9NRD8-1. The sequences were aligned by Clustal Omega Multiple Sequence Alignment from EMBL.

With the insights gained from the characterization of the EF‐domain, we sought to investigate how this domain activates the NOX5 catalytic core within the context of the full‐length protein. We used membranes extracted from *Escherichia coli* overexpressing csNOX5, which we found to be a most convenient production system for our biochemical and mutagenesis analysis. We first checked by western blot that all mutants were expressed in amounts comparable to the wild‐type (Fig. 9A). We then confirmed that wild‐type csNOX5 is activated by calcium. The enzymatic activity was barely detectable and gradually increased by adding calcium reaching a plateau at 1 mM CaCl_2_ (Fig. 9B). Furthermore, low or no calcium activities were detected for two N‐terminally truncated variants lacking the N‐lobe and the entire EF‐domain (Table 2).

**Table 2:** Activities of the EF‐binding region mutants of csNOX5. Data are the average of duplicates

csNOX5 construct	ΔAbs_550 nm·_s^‐1^	
	No CaCl_2_	1 mM CaCl_2_
Wild‐type	0.0032 ± 0.0014	0.0159 ± 0.0004
D611H	0.0018 ± 0.0001	0.0036 ± 0.0008
D631A	0.0018 ± 0.0003	0.0019 ± 0.0006
D631R	0.0027 ± 0.0010	0.0016 ± 0.0003
csNOX5 EF‐truncated^a^	0.0018 ± 0.0001	0.0025 ± 0.0003
csNOX5 N‐lobe truncated	0.0020 ± 0.0004	0.0035 ± 0.0003
Background^b^	0.0005–0.001	0.0005–0.001

a csNOX5 EF‐truncated comprises residues 209‐693 of csNOX5 and corresponds to a natural appearing variant of hNOX5, called NOX5ε, which lacks the EF‐domain

b Background is measured in membranes not expressing csNOX5.

To further validate our results, we next reconsidered previous work performed with peptides (rather than the full‐length protein or domains) which had suggested that the EF‐domain activates NOX5 by interacting with the so‐called ‘region EF‐binding domain’ (residues 638‐661 of hNOX5 isoform β and 611‐634 of csNOX5) in the DH‐domain 4, 16. In the csDH crystal structure, the region is disordered and solvent‐exposed 4. We further noticed that this segment contains two conserved aspartate residues (Asp611 and Asp631 in csNOX5) that are conserved in all NOXs with the exception for the nonregulated and constitutively active NOX4. Importantly, in hNOX2, these residues (Asp484, Asp501) are part of a region involved in the enzyme activation by the p47phox/p67phox cytosolic proteins 14, 22 (Fig. 9C). Given these observations, we prepared three csNOX5 mutants (D611H, D631R, D631A) and found that none of them is activated by calcium, also at high calcium concentrations (Table 2). Collectively, these data validated the essential role of the ‘region EF‐binding domain’ with a specific role played by the highly conserved aspartates.

**Figure 10:**
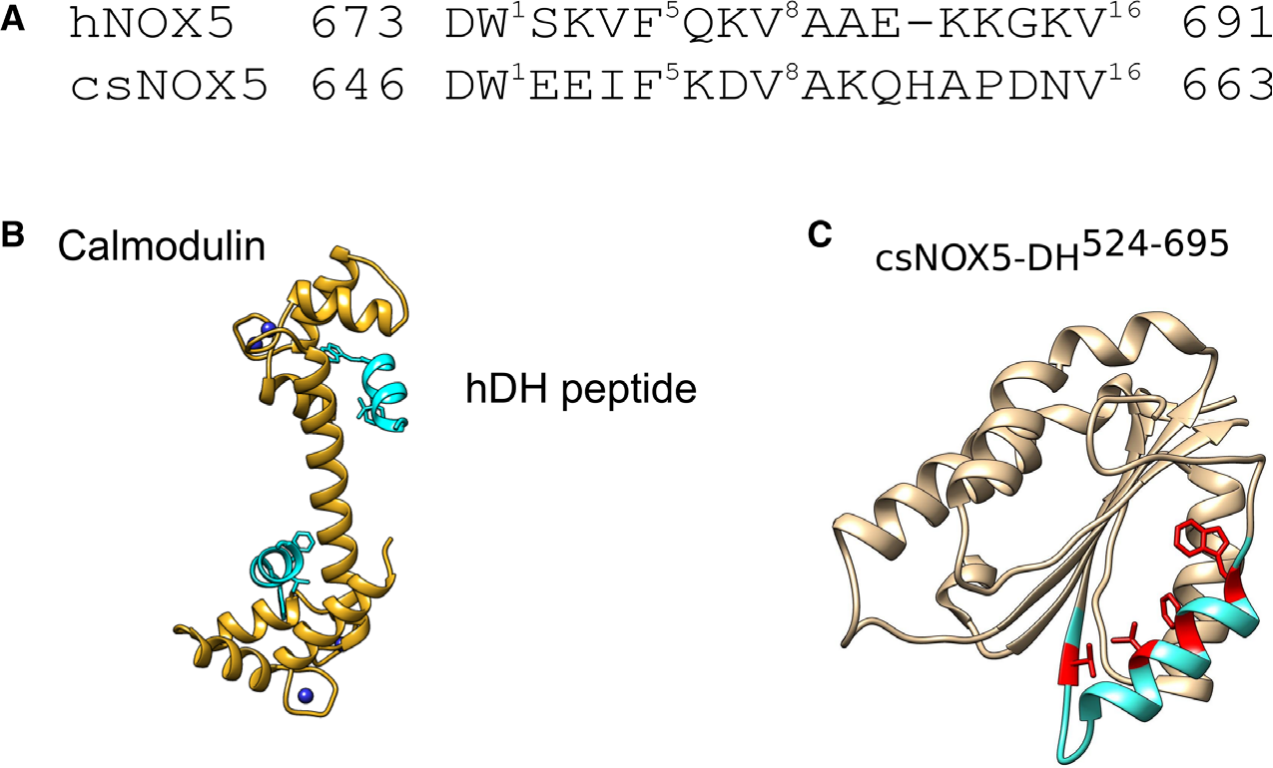
A physiologically unlikely binding mode of calmodulin to DH. (A) The predicted calmodulin‐binding region of hNOX5 is conserved in csNOX5. Bulky residues on positions 1, 5, 8, and 16 of the expected calmodulin‐binding loop correspond to Trp674, Phe678, Val681, and Val691 in hNOX5 and Trp647, Phe651, Val654, Val663 in csNOX5. Clustal Omega Multiple Sequence Alignment from EMBL was used for alignment (B) Crystal structure human calmodulin (golden) with four calcium ions (dark blue) bound to the putative calmodulin‐binding region of the DH‐domain of hNOX5 (residues 674‐685; light blue). Each lobe of the calmodulin is bound to one peptide through interactions with Trp674, Phe678, Val681, and Val691 of the DH peptide. Model images were displayed by UCSF Chimera. Structure deposited at PDB with code 6SZ5. (C) These residues on the putative calmodulin‐binding helix are, however, buried in the DH‐domain hydrophobic core as it can be seen in the crystal structure of csNOX5 DH‐domain (csNOX5: Trp647, Phe651, Val654, Val662).

We then investigated whether the activity of csNOX5 and of the deregulated mutants was affected by calmodulin, since this protein has been reported to increase hNOX5 activity under conditions of low calcium concentrations in which the enzyme cannot reach the maximum activity by its own 7. We found that calmodulin does not activate wild‐type or mutated csNOX5 at any calcium concentration (Table 3). This finding could reflect the absence of calmodulin in cyanobacteria, implying that no response to calmodulin should be expected for csNOX5. We then cocrystallized human calmodulin with a peptide corresponding to the putative calmodulin‐binding region located on the DH‐domain of hNOX5 (residues 674–685) 7. The crystal structure revealed that the peptide does bind to calmodulin 7. However, mapping the peptide onto the three‐dimensional structure of the DH domain showed that the residues involved in the interaction are buried in the domain hydrophobic core (Fig. 10). Binding of calmodulin to NOX5 would thereby require the unlikely and unfavorable unfolding of the DH‐domain. It thus appears that residues 674–685 4 of the human DH‐domain fortuitously contain a conserved pattern of hydrophobic residues that matches the calmodulin‐binding motif but this interaction is most unlikely to represent a bona fide site for DH‐calmodulin interactions.

**Table 3:** The role of calmodulin in NOX5 activation. The measurements were carried out in the presence of 1 mM CaCl_2_.

csNOX5 protein	ΔAbs_550 nm_·_s_^‐1^
Wild‐type	0.017
Wild‐type + calmodulin	0.016
D631A + calmodulin	0.002
D631R + calmodulin	0.002
D611H + calmodulin	0.002

## Discussion

The aim of this study was to characterize the structural basis of calcium binding in the EF‐domain of NOX5. The EF‐domain has a calmodulin‐like fold with four EF‐hand motifs. It is known that some EF‐hand proteins are able to fold also in the absence of calcium, as it is the case for calmodulin and *Lethocerus* troponin C 23. Others, such as plant calmodulin‐like protein 19 or neuronal calcium sensor‐1, are intrinsically unfolded and become folded only in the presence of calcium 24, 25. CD, NMR, and hydrogen‐deuterium exchange consistently demonstrated that the EF‐domain of NOX5 belongs to the second category: The apo protein presents elements of secondary structure but lacks definite tertiary structure, as reflected by the poor spectral dispersion of the NMR spectrum.

We also used NMR to validate the number of active EF‐hands of NOX5. This technique confirmed the presence of resonances at ~11 ^1^H p.p.m. and ~108 ^15^N in the ^1^H‐^15^N HSQC spectrum, known to correspond to glycine in position 6 of the canonical EF‐loops only when calcium is bound 19. We saw only three resonances at the expected chemical shifts, but a closer look to the sequence revealed that the first EF‐hand does not contain the conserved glycine in position 6 of the loop. EF1 belongs to the category of noncanonical or calpain‐like EF‐hands which have a different calcium coordination arrangement 6. Evidence obtained both from NMR and hydrogen‐deuterium exchange corroborated the notion that the EF‐hands 2, 3, and 4 are canonical motifs (calmodulin‐like), whereas EF1 is a noncanonical but yet calcium‐binding site 6, 17. We also experimentally verified that all EF‐hands of NOX5 are calcium specific, as 1 mM Mg^2+^ did not shift the apo NMR spectrum toward the holo spectrum. This seems contradictory with previous studies which reported Mg^2+^ binding to NOX5‐EF3 25. However, these studies used a higher ion concentration (*K*_a_ = 4 mM) and reported only minute changes of the structure 17, 26. It is thus possible that Mg^2+^ weakly bind NOX5‐EF3 but it cannot anyway trigger the holo state formation and enzyme activation.

We then investigated the calcium‐dependent conformational transition in each of the lobes separately. We found that the N‐lobe has considerable tertiary structures in both apo and holo states. The C‐lobe is instead disordered in the absence of calcium but acquires a structure in the holo state. Accordingly, a recent study reported that the C‐lobe is less stable than the N‐lobe but has a higher affinity for calcium 26. Consequently, our studies show that the C‐lobe drives the calcium‐induced conformational change of the EF‐domain of NOX5. We can thus hypothesize that the plasticity of the C‐lobe determines the behavior of the full domain. Other EF‐hand proteins such as human centrin 2 have a similar behavior: The N‐lobe of centrin 2 is compact and structured in the absence of calcium, while the C‐lobe has a calcium‐dependent fold for function‐related reasons. In centrin 2, the C‐lobe is responsible for partner binding and is the main determinant for the cellular regulatory role, whereas the N‐lobe binds the C‐lobe and helps oligomerization of the protein 27, 28. Even though these results need to be confirmed with proteins from the same organism, the notion that the C‐lobe acts as the regulatory domain is further corroborated by the observation that the C‐lobe seems to interact with the DH‐domain, while no interaction was detected for the N‐lobe. The function of the N‐lobe remains unclear but our mutagenesis data on a bacterial homologue of hNOX5 show that this lobe is needed for activation (Table 2). It is possible that the N‐lobe facilitates folding and/or calcium binding and/or interaction with the DH‐domain but only in the context of the full‐length protein.

To further understand the role of the EF‐domain in enzyme regulation, we studied the full‐length NOX5. To this aim, we used a bacterial orthologue of hNOX5, which was previously established as a good and convenient system to measure activities and perform structural studies. Earlier peptide‐based studies suggested that the EF‐domain activates NOX5 by interacting with the catalytic DH‐domain. When conserved residues in the predicted EF‐binding region of the DH‐domain (D611H, D631R, D631A) were mutated, the enzyme became calcium‐insensitive and virtually inactive. Interestingly, these data resonate with a study on NOX2, in which mutation of the equivalent residues (Asp484 and Asp500 in hNOX2) resulted in the defective translocation of the NOX2 partner p47phox 22. According to these observations, this segment of the DH‐domain is hereby strongly predicted to be critical not only for the activation of NOX5 but of all NOX proteins, notwithstanding that each of them has a distinct regulatory mechanism mediated by different activating protein partners.

In conclusion, our study clarifies the molecular bases of the regulation of NOX5 and opens new avenues to an understanding of the role of this class of enzymes in ROS regulation.

## Materials and methods

### Cloning, protein expression, and purification

The genes encoding csNOX5 (UNIPROT code K9WT99) and hNOX5 (isoform β, known also as v2; UNIPROT code Q96PH1-4) were purchased from Gene‐Script (Piscataway, NJ, USA). Single‐site mutations on csNOX5 were introduced by site‐direct mutagenesis using In‐fusion Cloning following the manufacturer’s instructions (In‐fusion Cloning; Takara, Kusatsu, Shiga Prefecture, Japan). Alignments were performed with Clustal Omega Multiple Sequence Alignment from EMBL and ESPrit3 29, 30

The EF‐domain of hNOX5 (residues 1–161) and the two isolated N‐ and C‐lobes (residues 1–77 and 78–161, respectively) were expressed as fusion proteins containing an N‐terminal Strep‐tag^®^ followed by a tobacco etch virus cleavage site. A stretch of three alanines corresponding to the Not I restriction site was introduced at the C terminus for cloning reasons. Expression of the ^15^N‐labeled or ^15^N,^13^C‐double‐labeled human EF‐domain, C‐ and N‐lobe proteins was carried out using *E. coli* strain BL21 cells (Novagen, Darmstadt, Germany) grown in minimal medium with M9 salts. Once the cells reached an OD_600_ of 0.6, protein expression was induced with 0.5 mM isopropyl‐β‐d‐thiogalactopyranoside at 17 °C for 16 h. Cells were collected by centrifugation and the pellet resuspended in ice in lysis buffer (50 mM Hepes pH 7, 100 mM NaCl, 10% (v/v) glycerol, 1 mM DTT, 1 mM PMSF). Cells were lysed by sonication and the cell debris removed by centrifugation. The supernatant was purified using a Strep column on an ÄKTA system (GE Healthcare, Chicago, IL, USA). The proteins were eluted with 50 mM Hepes pH 7.0, 200 mM NaCl, 1 mM DTT, and 3 mM d‐desthiobiotin. The sample was dialyzed overnight against 50 mM Hepes buffer at pH 7.0, 500 mM NaCl and 1 mM DTT while cleaved by His_6_‐tagged tobacco etch virus protease at room temperature. Sequential loading through Strep and Nickel columns was used to remove the cleaved Strep‐tag and the protease. The sample was finally gel‐filtered with a Superdex 75 column (GE Healthcare) equilibrated in 50 mM Hepes pH 7.0, 25 mM NaCl, 0.5 mM TCEP, 1 mM CaCl_2_ or 1 mM EGTA.

The *C. stagnale* EF‐domain (residues 1–180) was produced in a similar way using a tag‐free construct. The cell extract was purified using a HiTrap Phenyl Sepharose HP column in buffer 50 mM Hepes pH 7.6, 250 mM NaCl, 1 mM DTT, 5 mM MgCl_2_, 1 mM CaCl_2_, 5% (v/v) glycerol, 1 mM PMSF. Nonspecifically bound proteins were first removed in a high salt buffer (500 mM NaCl), and the EF‐domain was then eluted with 50 mM Hepes pH 7.5, 40 mM NaCl, 2 mM EGTA, 5% (v/v) glycerol, and 1 mM EGTA. However, the protein resulted to be systematically aggregated and prone to precipitation. The experiments with the *C. stagnale* DH‐domain were performed with a thermostable mutant (csDH^PWLELAAA^) which was expressed and purified as previously reported 4. The gene for human calmodulin (a kind gift from Susan Shao; LMB‐MRC, Cambridge, UK) was cloned in a pET15b vector (Novagen) without tag and purified following established protocols 31, 32.

Purified protein concentrations were estimated by Abs_280nm_ using 280 nm extinction coefficients of 53.400 m ^−1^·cm^−1^ for csDH^PWLELAAA^, 12.492 m ^−1^·cm^−1^ for human EF‐domain, 11.000 m ^−1^·cm^−1^ for the N‐lobe the human EF‐domain, 1.492 m ^−1^·cm^−1^ for C‐lobe of the human EF‐domain, 2.980 m ^−1^·cm^−1^ for human calmodulin. Purity was verified with SDS/PAGE electrophoresis.

csNOX5 proteins (wild‐type and D611H, D631A, D631R mutants) were expressed in *E. coli* BL21 (DE3) RP Plus (Novagen) as fusion protein containing an N‐terminal FLAG‐His_8_‐SUMO tag followed by a SUMO protease cleavage site. Cells were grown in 2xTY media at 37 °C until they reached an OD_600_ of 1.2. They were then induced with 0.3 mM isopropyl‐β‐D‐thiogalactopyranoside for 16 h at 17 °C. Cells were harvested, resuspended in 50 mM Hepes pH 7.5, 300 mM NaCl, 5% (v/v) glycerol, 1 µm leupeptin, 1 µm pepstatin, and 1 mM PMSF, and lysed by sonication. After a first centrifugation at 5000 ***g*** for 5 min, the supernatants were recovered and centrifuged again at 56 000 ***g*** for 80 min. Pellets were resuspended in 50 mM Hepes pH 7.5, 300 mM NaCl, 5% (v/v) glycerol, incubated for 30 min with 30 μm FAD and 30 μm Hemin, and centrifuged again at 56 000 ***g*** for 80 min. The final pellets were resuspended in 50 mM Hepes pH 7.5, 300 mM NaCl, 20% (v/v) glycerol. Membrane protein expression levels and concentrations were by the Biuret assay and western blots. Heme incorporation was measured by the absorbance at 414 nm.

### Western blotting

Proteins were separated by 12% (w/v) SDS/PAGE and blotted onto PVDF (Bio‐Rad, Hercules, CA, USA). Membranes were blocked in PBS containing 5% milk and incubated with an anti‐FLAG HRP‐conjugated monoclonal antibody (Sigma, St. Louis, MO, USA).

### Secondary structure studies

CD measurements were performed on a Jasco J‐715 spectropolarimeter (Jasco UK Ltd, Great Dunmow, UK) equipped with a cell holder thermostated by a PTC‐348 Peltier system. Far‐UV CD measurements were performed at 27 °C in 10 mM Tris‐H_2_SO_4_ buffer at pH 7.0 and 0.5 mM TCEP after pretreatment with a Chelex^®^ 100 resin. Proteins were used at a final concentration of 0.15 mg·mL^−1^. The spectra were recorded in fused silica cuvettes (Hellma, Milano, Italy) of 1 mM path length. Measurements were done in duplicates.

### NMR spectroscopy and sequential assignment

^1^H‐^15^N HSQC, HNCACB, and HNcoCANH spectra were recorded on 700, 800, and 950 MHz Bruker spectrometers equipped with cryo‐probes. The temperature was set at 27 °C to study the apo/holo states and at 20 °C to probe the interaction with the DH. Protein samples (200 µm) of the C‐terminal domain were in a 50 mM Hepes buffer at pH 7.0, 25 mM NaCl, 0.5 TCEP with the addition of 1 mM of CaCl_2_ to obtain the holo states. The interactions between the EF‐domain of hNOX5 and the DH‐domain of csNOX5 were probed by mixing the labeled N‐ or C‐lobes of the EF‐domain (40 µm) with unlabeled DH‐domain (80 µm). Water was suppressed by the WATERGATE pulse sequence. Spectra were processed with NMRPipe‐based scripts and NMRDraw 33. Assignment of the NMR spectrum was performed with the software CcpNmr Analysis 2.4 34 and deposited to the BMRB database (BMRB number: 50093).

### Superoxide measurements on membranes containing csNOX5 wild‐type and mutants

The enzymatic activity of the wild‐type and mutant csNOX5 was evaluated with csNOX5‐containing membrane fractions and cytochrome c (Sigma) as a superoxide scavenger. Membranes of csNOX5 wild‐type or mutants (80 μg) were added to a cuvette containing 2 mM sodium azide, 100 μm cytochrome c, and 200 μm FAD. NADPH (final concentration 500 μm) was added to the solution to start the reaction. Superoxide generation was monitored by following the increase in absorbance at 550 nm caused by the reduction of cytochrome c by superoxide. To evaluate the calcium‐dependent activation, 1 mM CaCl_2_ was added to the sample. Negative controls were performed with membranes from *E. coli* cells expressing the csNOX5 mutant lacking the EF‐domain or not expressing csNOX5 at all. The initial rates for cytochrome c reduction were calculated by measuring the ΔAbs at 550 nm within the first 20 s after NADPH addition. To evaluate the influence of calmodulin on the activity, csNOX5 membranes were incubated for 5 min with 10 µm calmodulin, a tenfold excess over the estimated concentration of csNOX5 in the membranes 35.

### Hydrogen‐deuterium exchange

Nepenthesin‐1 acid protease column (N1) was used for peptide mapping of human EF. Three hundred picomoles of the human EF‐domain was mixed in 1 : 1 ratio with 1 m glycine at pH 2.3, 8 m urea and injected on a N1 column placed in an ice box. Protein digestion and desalting of the generated peptides (peptide Microtrap column; Optimized Technologies, OR, USA) were performed for 3 min at a flow rate 100 µL·min^‐1^ using isocratic pump delivering solvent composed of 0.4% formic acid in water. After 3 min, the resulting peptides were separated on a C18 reversed phase column (ZORBAX 300SB‐C18 3.5 μm, 0.5 × 35 mM; Agilent, Santa Clara, CA, USA) with a linear gradient 10–30% B in 18 min, where solvent A was 2% acetonitrile/ 0.4% formic acid in water and solvent B 95% acetonitrile/ 5% water/ 0.4% formic acid. Both the desalting and analytical columns were placed in an ice box. Detection of the peptides was performed by a 15T solariX XR FT‐ICR mass spectrometer (Bruker Daltonics, Billerica, MA, USA) operating in positive MS/MS mode. Data were processed by dataanalysis 4.2 software (Bruker Daltonics). Identification of peptides was done by the MASCOT search engine against a database containing sequence of human EF.

Hydrogen‐deuterium exchange was started by 10‐fold dilution of the human EF‐domain (50 mM Hepes at pH 7.0, 100 mM NaCl, and 1 mM EGTA or 5 mM CaCl_2_) in a deuterated buffer containing 50 mM Hepes at pH/pD 7.0, 100 mM NaCl, and 1 mM EGTA or 5 mM CaCl_2_. Fifty‐microliter aliquots (100 pmols) were taken after 20 s, 2 min, 20 min, and 2 h of incubation in deuterated buffer and quenched by 50 µL of 1 m glycine at pH 2.3, 8 m urea, and fast‐freezing in liquid nitrogen. Aliquots were analyzed using the same system as described above. Mass spectrometer was operated in positive MS mode. Spectra of partially deuterated peptides were processed by data analysis 4.2 (Bruker Daltonics) and by in‐house program DeutEx.

### Crystallization and structure determination

Peptides corresponding to the putative calmodulin‐binding of the human DH ‐domain 4, 16 were custom‐synthesized with N‐terminal acetylation and C‐terminal amidation to avoid charge interferences (Chinepeptide Co, Shanghai, China). Initial crystallization experiments of human calmodulin in complex with the hNOX5 peptides were carried out at 20 °C using Oryx8 robot (Douglas Instruments, Hungerford, UK) and the sitting‐drop vapor‐diffusion technique. The crystallization droplets were formed by mixing 0.2 μL of the protein solution consisting of 1078 µm calmodulin, 2800 µm peptides in 30 mM Tris/HCl at pH 7.4, 2.5 mM CaCl_2_ and 2.5% (v/v) glycerol, and 0.2 μL of the reservoir from commercial screens (JCGS core suite I, II, III, and IV from Qiagen, Hilden, Germany and Morpheus from Hampton, Aliso Viejo, CA, USA). Crystals of human calmodulin in complex with the peptide 673–691 of human DH‐domain grew after 1 week under conditions containing 12.5% (w/v) PEG 1000, 12.5% (w/v) PEG 3350, 12.5% (v/v) MPD, 0.2 m l‐Na‐glutamate, 0.2 m alanine‐racemic, 0.2 m glycine, 0.2 m lysine‐racemic, 0.2 m serine‐racemic, 0.1 m MES/imidazole pH 6.5. Crystals were harvested and flash‐frozen in liquid nitrogen. Data were collected at the automatic MASSIF‐1 beamline in the European Synchrotron Radiation Facility (Grenoble, France). Datasets were indexed and integrated with XDS 36 and scaled with aimless (CCP4suite) 37. The structure of calmodulin was solved by molecular replacement using two copies of the N‐terminal lobe of calmodulin in a complex with STRA6 peptide (amino acids 16–71, PDB code 5k8q) using Phaser Molecular Replacement and ARP/wARP classic (CCP4suite) 37 (Table 4). Coot 38 was used for model building and REFMAC for refinement 39. Model images were done UCSF Chimera 40. The coordinate and structure factors were deposited with the PDB under accession code 6SZ5.

**Table 4:** Data collection and phasing statistics^a^.

Space group	P 2_1_ 2_1_ 2_1_	*R*_merge_ a	0.185 (0.98)	*R*‐factor (%)	21.9
Cell dimensions *a*, *b*, *c* (Å)	24.76, 60.84, 102.44	*I*/σ(*I*)^a^	6.5 (1.1)	Free *R*‐factor (%)	29.4
Wavelength (Å)	1.07227	CC1/2^a^	0.99 (0.53)	Root‐mean‐square bonds (Å)	0.010
Resolution (Å)^a^	2.2	Completeness (%)^a^	99.7 (100)	Root‐mean‐square bonds (°)	1.4
Number of reflections	8064	Redundancy^a^	6.0 (6.3)		

a Values in parentheses are for highest‐resolution shell.

## Conflict of interest

The authors declare no conflict of interest.

## Author contributions

EMF collected and analyzed the data and wrote the initial draft of the manuscript, ST helped in characterization of mutants and calmodulin, AS assisted the NMR processing, LM provided support to the acquisition and analysis of the CD data, PP performed the HDX exchange measurements, FM and AM cosupervised the overall project, AP designed the layout of the research and wrote the manuscript.
